# OLS Client and OLS Dialog: Open Source Tools to Annotate Public Omics Datasets

**DOI:** 10.1002/pmic.201700244

**Published:** 2017-10-09

**Authors:** Yasset Perez‐Riverol, Tobias Ternent, Maximilian Koch, Harald Barsnes, Olga Vrousgou, Simon Jupp, Juan Antonio Vizcaíno

**Affiliations:** ^1^ European Molecular Biology Laboratory European Bioinformatics Institute (EMBL‐EBI), Wellcome Trust Genome Campus Hinxton Cambridge UK; ^2^ Proteomics Unit, Department of Biomedicine University of Bergen Bergen Norway; ^3^ Computational Biology Unit, Department of Informatics University of Bergen Bergen Norway

**Keywords:** data annotation, omics datasets, ontologies, open source software

## Abstract

The availability of user‐friendly software to annotate biological datasets and experimental details is becoming essential in data management practices, both in local storage systems and in public databases. The Ontology Lookup Service (OLS, http://www.ebi.ac.uk/ols) is a popular centralized service to query, browse and navigate biomedical ontologies and controlled vocabularies. Recently, the OLS framework has been completely redeveloped (version 3.0), including enhancements in the data model, like the added support for Web Ontology Language based ontologies, among many other improvements. However, the new OLS is not backwards compatible and new software tools are needed to enable access to this widely used framework now that the previous version is no longer available. We here present the OLS Client as a free, open‐source Java library to retrieve information from the new version of the OLS. It enables rapid tool creation by providing a robust, pluggable programming interface and common data model to programmatically access the OLS. The library has already been integrated and is routinely used by several bioinformatics resources and related data annotation tools. Secondly, we also introduce an updated version of the OLS Dialog (version 2.0), a Java graphical user interface that can be easily plugged into Java desktop applications to access the OLS. The software and related documentation are freely available at https://github.com/PRIDE-Utilities/ols-client and https://github.com/PRIDE-Toolsuite/ols-dialog.

Modern systems biology and bioinformatics approaches rely on the integration of large amounts of potentially disparate data, coming from multiple biological samples, being generated by different techniques (e.g. omics approaches) and instrumentation.^1^ The management, integration, and reuse of data require an accurate and comprehensive capture of the associated metadata, including details such as the description of the samples, the experimental design, the processing steps, and the new biological evidences and claims, among others.^2^ Therefore, proper and consistent annotation of the generated data is essential in order to make sense of all the information. Ontologies and controlled vocabularies (CVs) have demonstrated their usefulness in enabling the consistent annotation of large volumes of complex data in the life sciences.^3^ Note that in the following text, we will for simplicity use the term “ontology” to refer to both “ontologies” and “CVs”.

Since 2006, the Ontology Lookup Service (OLS, http://www.ebi.ac.uk/ols) at the European Bioinformatics Institute (EMBL‐EBI) has provided a popular and centralized framework to query, browse and navigate biomedical ontologies in the obo format,^4^ removing the need to search individual websites (for particular ontologies) or having to parse flat‐files available elsewhere. The ontologies are maintained by domain experts in the respective fields.^3^ The new iteration of the OLS (version 3.0, originally released on May 2016) constitutes a complete redevelopment of the framework, including improvements in the data model e.g. added support for Web Ontology Language (OWL)‐based ontologies, an increase in the number of ontologies covered, as well as multiple enhancements in its web and programmatic interfaces, and in the underlying backend. By June 2017 the OLS integrates 191 ontologies, which correspond to around 4.9 million unique ontology terms. The biggest change in the new OLS has been to move from an eXtensible Markup Language based Simple Object Access Protocol Application Program Interface (API) to a JavaScript Object Notation based REpresentational State Transfer one that requires changes to the client application written for the old system.

Here we present the OLS Client, an open source Java API, providing a comprehensive functionality to programmatically query, browse and retrieve all information from the OLS. It can handle all the main data types in the OLS, ranging from ontology terms and annotations to graph ontology term relationships such as *child* and *parent* terms. In addition, we present an update of the OLS Dialog (version 2.0), a Java graphical user interface (GUI) built on top of the OLS Client that can be easily plugged into Java desktop applications. To the best of our knowledge, OLS Client and OLS Dialog 2.0 are the first open source Java APIs available for the new version of the OLS.

The OLS Client library (https://github.com/PRIDE-Utilities/ols-client) provides a unified access interface to ontologies and all related information in the OLS (Figure 1). The interface provides methods to access and retrieve information on each ontology term including annotations, synonyms and all types of identifiers. The API is identifier independent. This represents one of the most convenient functionalities of the OLS Client given that each term can be directly retrieved using their corresponding identifiers, including the Uniform Resource Identifier (URI) or Compact URI (CURIE). Following the OLS 3.0 data structure, the OLS Client data model represents a well‐connected graph where every term in each ontology contains a list of *parent* and *child* terms. The API is composed of two functional components: (i) the data models, incorporating all data structures (terms, identifiers, ontologies, annotations and synonyms); and (ii) the OLS Client query interface, providing a set of functionalities to query, browse and retrieve the ontology's related information. The data models are used by the query interface to represent the retrieved data, as shown in the example code snippet below:

*OLSClientolsClient = new OLSClient(new OLSWsConfigProd());*
*Term term = olsClient.getTermById(new Identifier(“MS:1001767”, Identifier.IdentifierType.OBO), “MS”)*.

**Figure 1:**
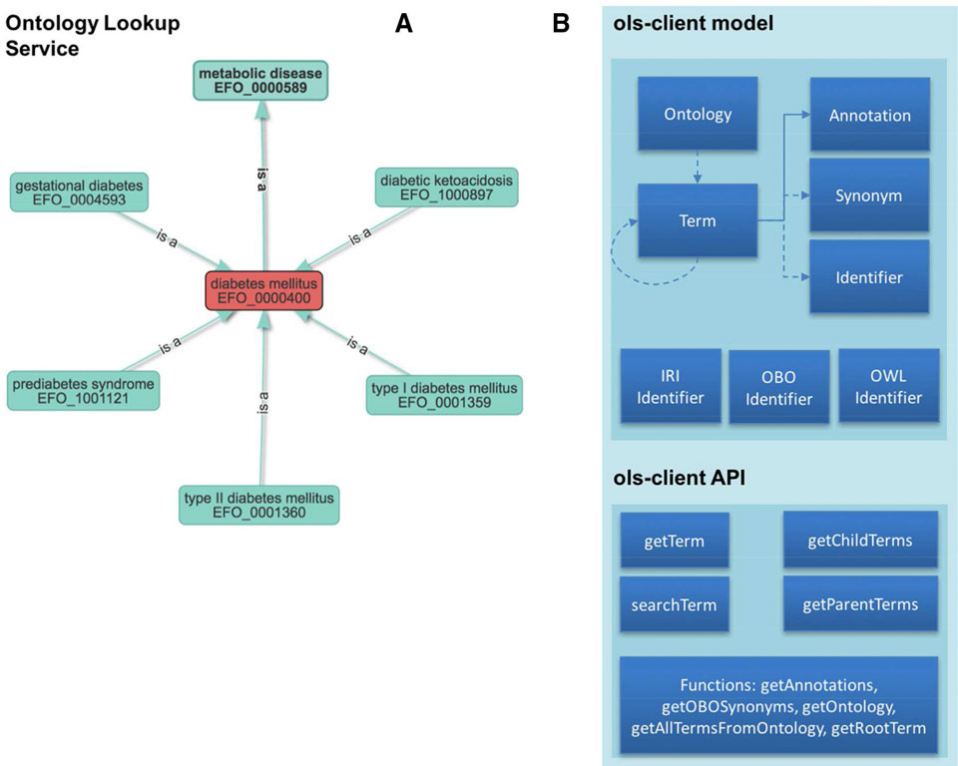
Overview of the design of the OLS Client: (A) Graph structure of the ontology terms relations for EFO Ontology and (B) Data structure of OLS‐Client library to represent and handle the OLS information.

**Efficient mining of millions of ontology terms**: Each ontology can potentially contain thousands of terms in well‐connected graphs. This complexity prompts a new challenge when users want to mine one specific ontology. Then, OLS Client implements an efficient mechanism to retrieve all terms in an ontology graph using pagination, and recursive querying of the OLS web API. Every time a specific ontology term is requested, the complete list of all the identifiers of all its child terms is retrieved. In addition, the API can obtain recursively all the term's information split into chunks. Furthermore, the OLS Client provides simple search functionality of the OLS, as shown in the example code snippet below and in the following section:

*OLSClientolsClient = new OLSClient(new OLSWsConfigProd());*
*List<Term> terms = olsClient.getTermsByName(“modification”, “ms”, true)*.

**OLS Dialog 2.0**. A new version of the OLS Dialog^5^ (https://github.com/PRIDE-Toolsuite/ols-dialog) has also been implemented, providing a GUI that can be plugged into any Java standalone application used for data annotation purposes. The OLS Dialog greatly simplifies the usage of the OLS Client since it does not require any additional knowledge about the OLS web services or the various ontology data formats. The OLS Dialog provides four different search strategies (Figure 2): (i) “Term Name Search”, which locates a term by a (partial) match to the searched text; (ii) “Term ID Search”, which selects a term by its accession number; (iii) “Browse Ontology” enables users to browse the selected ontology tree structure to select the desired term; and (iv) the “PSI‐MOD Mass Search”, which uses the PSI‐MOD ontology 6 and UNIMOD to select protein modifications using the delta mass corresponding to a given modification (Fig. 2). The former more specific search functionality is used for instance in the ProteomeXchange (PX) Submission tool,^7^ used by most submitters to the widely‐used PRIDE database (as part of PX). In all cases, when users select a concrete term, all the associated details will be presented in the “Term Details” table in the GUI, including its identifier, annotations and synonyms. The OLS Client is part of PRIDE‐Utilities^8^ and OLS Dialog is part of the PRIDE Inspector Toolsuite^9^ a set of Java components that can be reuse in proteomics Java applications.

**Figure 2:**
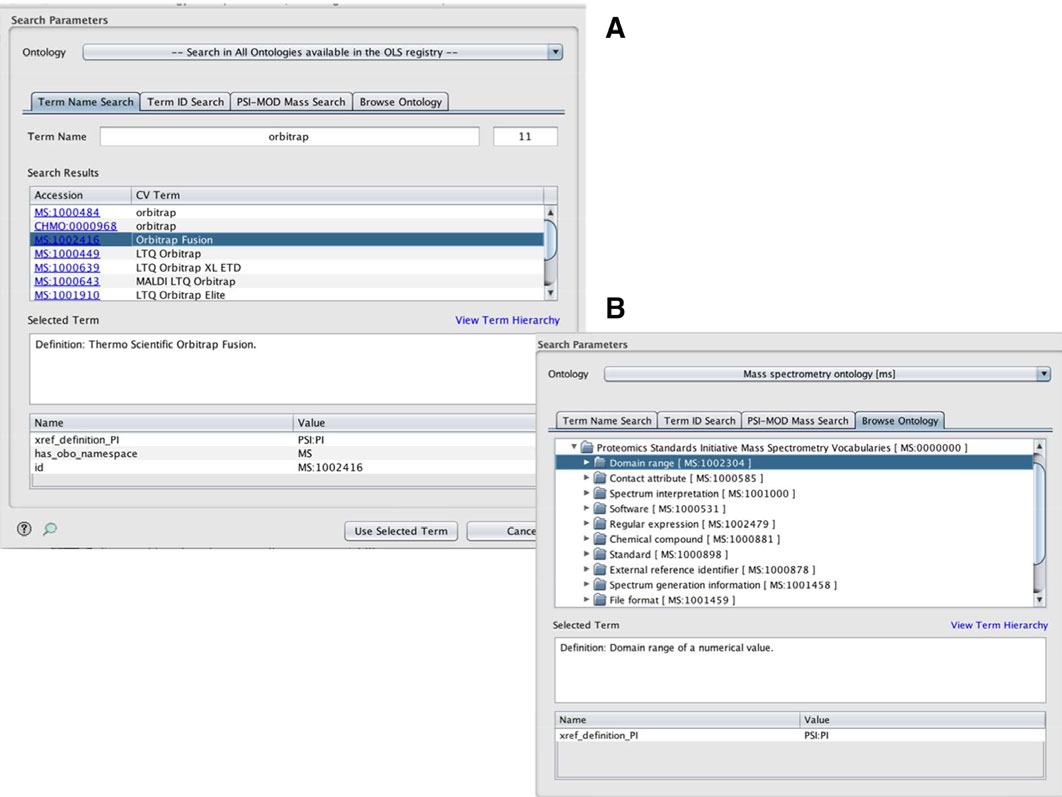
OLS Dialog main interfaces: (A) Search functionalities include searching by the name of the ontology term, the identifier of the term, or the PTM delta mass; while (B) Browse Ontology enables browsing across all the terms in the OLS to locate the desired term.

In conclusion, it is important to note that, at the moment of writing, several popular resources and tools are already using OLS Client as annotation source, including resources such as IntAct,^10^ OmicsDI,^11^ BioModels,^12^ and the Reactome Pathway Annotation Tool,^13^ or stand‐alone tools (which use OLS Dialog on top) such as PeptideShaker,^14^ the Laboratory Information Management System (LIMS) colims (https://github.com/compomics/colims) or the already mentioned PX Submission Tool (for a complete list see Table 1). The widespread use of the library ensures its stability, continued development, and community support. The OLS Client library and OLS Dialog (including the related documentation) are freely available and released under Apache 2.0 license at https://github.com/PRIDE-Utilities/ols-client and https://github.com/PRIDE-Toolsuite/ols-dialog, respectively.

**Table 1:** Software and resources using OLS Client and/or OLS Dialog (by June 2017)

Name	Description	URL	Tools
ProteomeXchange Submission Tool^7^	Stand‐alone submission tool for the PRIDE database	https://github.com/proteomexchange/px-submission-tool	OLS Client OLS Dialog
Reactome Annotation Tool^13^	Pathway annotation tool	http://www.reactome.org	OLS Client
IntAct^10^	Curated molecular interactions database	http://www.ebi.ac.uk/intact	OLS Client
PeptideShaker^14^	Search engine independent platform for the interpretation of proteomics identification results	http://compomics.github.io/projects/peptide-shaker	OLS Client
Omics Discovery Index^11^	A multi‐omics dataset discovery resource	http://www.omicsdi.org	OLS Client
Colims	A LIMS system to automate and expedite proteomics data management, processing and analysis	http://compomics.github.io/projects/colims	OLS Client OLS Dialog
BioSamples(7)	Resource that stores and supplies descriptions and metadata about biological samples	https://www.ebi.ac.uk/biosamples	OLS Client
CySBML(8)	Cytoscape plugin for importing and visualizing SBML annotations	https://sourceforge.net/projects/cysbml	OLS Client
BioModels Database^12^	BioModels Database is a repository of computational models of biological processes.	http://www.ebi.ac.uk/biomodels-main	OLS Client

**Abbreviations**
API: application programming interface
CURIE: Compact URI
CV: controlled vocabulary
GUI: graphical user interface
LIMS: Laboratory Information Management System
OLS: Ontology Lookup Service
OWL: Web Ontology Language
PX: ProteomeXchange
URI: Uniform Resource Identifier

## Conflict of Interest

The authors have declared no conflict of interest.
